# Cervical sagittal alignment in adolescent high dysplastic developmental spondylolisthesis: how does the cervical spine respond to the reduction of spondylolisthesis?

**DOI:** 10.1186/s13018-020-01762-y

**Published:** 2020-07-06

**Authors:** Xinhu Guo, Weishi Li, Zhongqiang Chen, Zhaoqing Guo, Qiang Qi, Yan Zeng, Chuiguo Sun, Woquan Zhong

**Affiliations:** Department of Orthopaedics, Peking University Third Hospital, No.49 Huayuan Bei Road, Beijing, 100191 China

**Keywords:** High dysplastic developmental spondylolisthesis, Developmental spondylolisthesis, Lumbosacral kyphosis, Cervical alignment, Cervical lordosis, Adolescent, Cervical kyphosis

## Abstract

**Background:**

Although pelvic and related parameters have been well stated in lumbar developmental spondylolisthesis, cervical sagittal alignment in these patients is poorly studied, especially in high dysplastic developmental spondylolisthesis (HDDS). The purpose of this study is to investigate the sagittal alignment of the cervical spine in HDDS and how the cervical spine responds to reduction of spondylolisthesis.

**Methods:**

Thirty-three adolescent patients with lumbar developmental spondylolisthesis who received preoperative and postoperative whole-spine x-rays were reviewed. They were divided into the HDDS group (*n* = 24, 13.0 ± 2.2 years old) and the low dysplastic developmental spondylolisthesis (LDDS) group (*n* = 9, 15.6 ± 1.9 years old). Spinal and pelvic sagittal parameters, including cervical lordosis (CL), were measured and compared between groups. In the HDDS group, the postoperative parameters were measured and compared with those before surgery.

**Results:**

HDDS group had a higher proportion of cervical kyphosis (70.8% vs. 22.2%, *P* = 0.019), and there was a significant difference in CL between the two groups (− 8.5° ± 16.1° vs. 10.5° ± 11.8°, *P* = 0.003). CL was correlated with the Dubousset’s lumbosacral angle (Dub-LSA), pelvic tilt (PT), and thoracic kyphosis (TK). In the HDDS group, CL in patients with a kyphotic cervical spine was significantly improved after reduction of spondylolisthesis (− 16.4° ± 5.9° vs. − 3.6° ± 9.9°, *P* < 0.001). In the HDDS group, 46% (6/13) of the patients with postoperative Dub-LSA < 90° still had sagittal imbalance (sagittal vertical axis, [SVA] > 5 cm), while no sagittal imbalance was observed in patients with postoperative Dub-LSA > 90° (46% [6/13] vs*.* 0% [0/11], *P* = 0.016).

**Conclusions:**

HDDS can lead to cervical kyphosis through a series of compensatory mechanisms. Reduction of spondylolisthesis and correction of lumbosacral kyphosis may correct the cervical kyphosis and normalize the overall spinal sagittal profile. Correction of Dub-LSA to above 90° might be used as an objective to better improve the sagittal alignment of the spine.

## Background

In the Marchetti-Bartolozzi classification, developmental spondylolisthesis is classified into high dysplastic developmental spondylolisthesis (HDDS) and low dysplastic developmental spondylolisthesis (LDDS) according to the degree of dysplasia [1]. HDDS is relatively rare, mainly occurring in teenagers and children and involving L5/S1, and is characterized by major deficiencies of the bone structure (such as dysplasia or malformation of the L5/S1 facets, lamina absence, spina bifida, domed sacral endplate, and trapezoidal L5 vertebral body), and vertebra slippage often leads to lumbosacral kyphosis [1, 2]. Therefore, HDDS is more progressive. LDDS is more common and differs from HDDS in that the L5 bodies remain rectangular, and the sacral upper endplate is preserved without lumbosacral kyphosis [1]. Many studies have reported that young patients with developmental spondylolisthesis often exhibit abnormal pelvic sagittal parameters as well and are more prone to sagittal imbalance [2–5]. However, majority of these studies did not distinguish between patients with HDDS and those with LDDS and did not consider cervical sagittal alignment. In clinical practice, we have observed that some HDDS patients showed cervical kyphosis, and that kyphosis was corrected spontaneously by reduction of spondylolisthesis. To date, whether HDDS is related to cervical kyphosis has not been elucidated. Therefore, we performed this study to investigate the sagittal alignment of the cervical spine in patients with HDDS and how the cervical spine responds to the reduction of spondylolisthesis.

## Methods

### Study population

This is a retrospective study. Adolescent patients diagnosed with developmental spondylolisthesis between April 2008 and April 2019 at our department were followed up and their data were analyzed. The inclusion criteria were as follows: (1) age< 18 years old when admitted to the hospital; (2) a diagnosis of L5-S1 developmental spondylolisthesis according to Marchetti-Bartolozzi’s criteria: some degree of congenital abnormality (dysplasia) of the posterior elements at the involved segments [1]; (3) availability of preoperative and postoperative standing whole-spine x-ray images; and (4) an indication for surgical treatment determined by the treating physician. Subjects with previous spinal surgery or with combined spinal trauma or congenital thoracic/cervical deformity were excluded from this study.

A total of 33 adolescent patients with lumbar developmental spondylolisthesis were included. They were divided into HDDS and LDDS groups according to Mac-Thiong and Labelle’s criteria [2]. There were 24 patients in the HDDS group (9 with grade II, 8 with grade III, 5 with grade IV, and 2 with grade V) and 9 patients in the LDDS group (6 with grade I and 3 with grade II), which was utilized as the control group.

### Radiographic measurement

The postoperative whole-spine x-rays’ taken time ranged from 3 to 100 months after surgery, and the median taken time was 7.5 months, and the mean taken time was 26.3 ± 31.9 months. A similar radiological protocol was used for all patients. The lateral radiographs were taken with the patient placed in an erect, comfortable stance and the knees fully extended. For the upper limbs, their arms were held in forward flexion or extended and resting on an arm support. The patients were instructed to keep a horizontal gaze to reduce inaccuracy caused by head motion. The anteroposterior radiographs were taken with the arms hanging freely at the side. General information was gathered, and spinal and pelvic sagittal parameters before and after the operation were measured. We used a custom computer application (PACS, GE Electrics) to measure the angles and distances. All parameters were measured twice by the first author with a month interval, and the average of the results was recorded. The parameters and measurement methods are as follows.

**Fig. 1:**
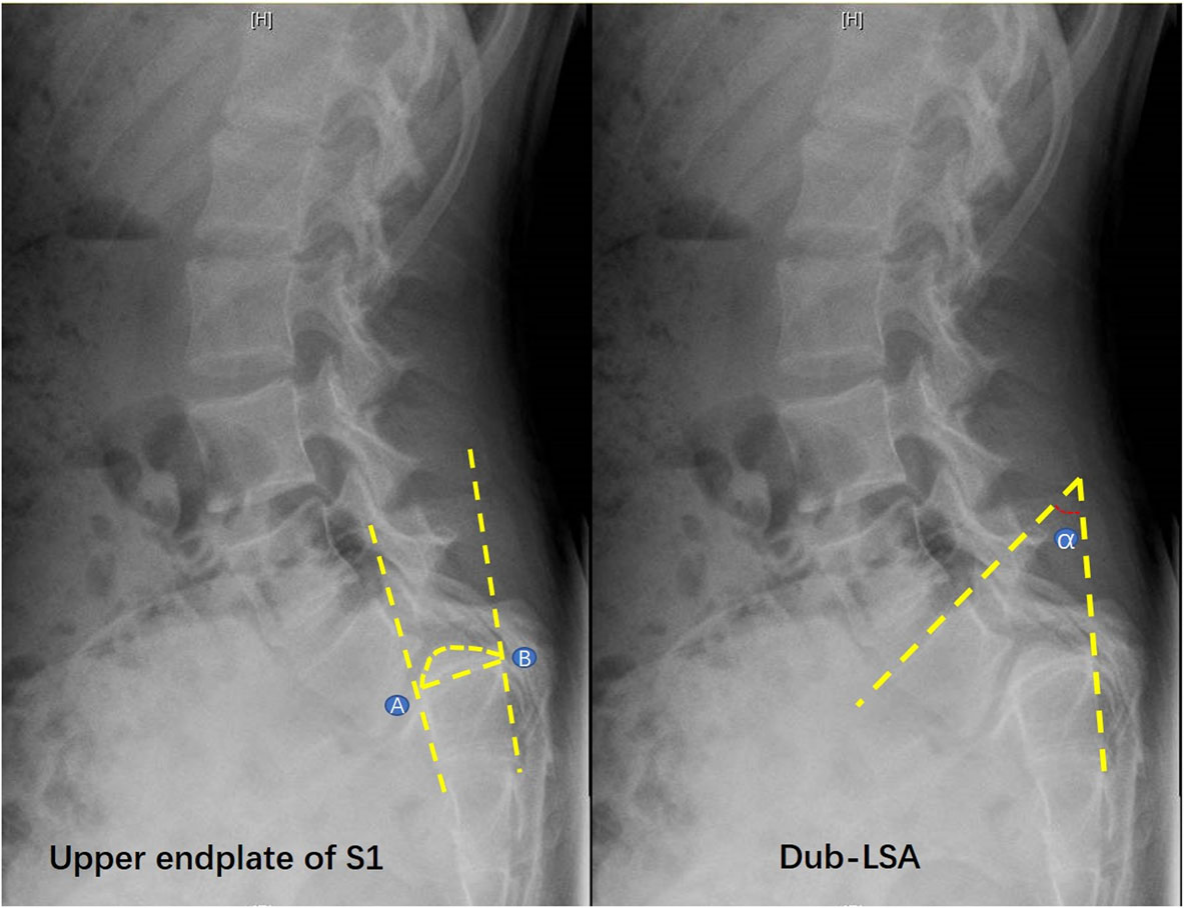
Measurement of the S1 upper endplate and Dub-LSA. In the left image, AB represents the upper endplate of S1 with a doming change; in the right image, α represents Dub-LSA

Evaluation of the slippage: (1) For patients with sacral doming, it can be difficult to perform precise geometric measurements involving the S1 endplate. In that case, the following technique can be used (Fig. 1) [1, 2]: two best-fit lines are drawn, one along the anterior and one along the posterior border of the sacrum; a third line is then drawn between the two tangent points of the first two lines and the doming; and the third line is considered the S1 endplate. (2) The degree of slip is measured by the Meyerding method and slip percentage. (3) The Dubousset lumbosacral angle (Dub-LSA) is used to evaluate the lumbosacral kyphosis and is described as the angle between the L5 upper endplate and the posterior border line of S1 vertebrae [6]. A Dub-LSA less than 90° is considered significant lumbosacral kyphosis. A smaller Dub-LSA represents heavier lumbosacral kyphosis.

Measurement of spinal and pelvic sagittal parameters: (1) Pelvic incidence (PI) is defined as the angle between a line joining the center of the upper endplate of S1 to the axis of the femoral heads and a line perpendicular to the upper endplate of S1. (2) Pelvic tilt (PT) is defined as the angle between the vertical line and a line drawn from the center of the upper endplate of S1 to the axis of the femoral heads. (3) Sacral slope (SS) is defined as the angle between the upper endplate and the horizontal line. (4) Lumbar lordosis (LL) is defined as the angle between the upper endplate of L1 and the lower endplate of L5, with a positive value indicating lordosis and a negative value indicating kyphosis. (5) Thoracic kyphosis (TK) is defined as the angle between the upper endplate of T4 and the lower endplate of T12, with a positive value indicating kyphosis and a negative value indicating lordosis. (6) Using the posterior tangent method, cervical lordosis (CL) is defined as the angle between the posterior border lines of the C2 vertebral body and the C7 vertebral body, with a positive value indicating lordosis and a negative value indicating kyphosis [7]. (7) Sagittal vertical axis (SVA) is defined as the distance between the plumb lines dropped from the center of the C7 vertebral body and the posterior-superior aspect of the S1 vertebral body. (8) Pelvic version was classified into balanced pelvis or unbalanced pelvis according to Hresko et al. [8]. Groups were divided by a line according to the following equation: SS = (0.844835 × PT) + 25.021 [8].

### Surgical methods

In the HDDS group, L5 resection with L4-S1 fixation and fusion was performed in one patient with grade V spondylolisthesis, complete or partial reduction of L5 with L4-S1 fixation and fusion was performed in 15 patients, and complete or partial reduction of L5 with L5-S1 fixation and fusion was performed in 8 patients. In the LDDS group, reduction of L5 with L5-S1 fixation and fusion was performed in 6 patients, and L5 pars repair was performed in 3 patients. We used posterior-only approach for all the cases except the L5 resection case, in which we used the combined anterior-posterior approach.

### Statistical analysis

Independent sample *t* tests were used to compare normally distributed data between the HDDS and LDDS groups. For non-normally distributed data, the Mann-Whitney rank sum test was adopted. Paired sample *t* tests were used to compare the preoperative and postoperative data. Pearson’s correlation analyses were used to analyze linear relationships between two parameters. The *χ*^2^ test was used to compare rates. SPSS version 21.0 (IBM Corporation, Armonk, NY, USA) was used for statistical analysis. The *α* value was set at 0.05.

## Results

The comparative results between the HDDS and LDDS groups are shown in Table 1. The age, Dub-LSA, SS, TK, and CL of the HDDS group were significantly smaller than those of the LDDS group, while the female ratio, slip percentage, PI, PT, and ratio of cervical kyphosis were significantly higher in the HDDS group than in the LDDS group. A higher proportion of patients in the HDDS group had sagittal imbalance (SVA > 5 cm) than that in the LDDS group, but there was no significant difference (41.7% [10/24] vs. 11.1% [1/9], *P* = 0.205). Pearson’s correlation tests were used to analyze the correlations among these parameters, and the results showed that CL had a strong correlation with TK (*r* = 0.683, *P* < 0.001), moderate correlations with Dub-LSA (*r* = − 0.446, *P* = 0.009) and PT (*r* = − 0.592, *P* < 0.001), and a weak correlation with PI (*r* = − 0.346, *P* = 0.048) (Table 2).

**Table 1:** Comparison of the parameters between the HDDS and LDDS groups

		HDDS group (*n* = 24)	LDDS group (*n* = 9)	*P* value
Sex	Male	3(12.5%)	8(89%)	< 0.001* (Fisher’s exact test)
	female	21(87.5%)	1(11%)	
Age (year)		13.0 ± 2.2	15.6 ± 1.9	0.005*
Slip percentage (%)		63.7 ± 25.5	26.0 ± 10.5	< 0.001*
Dub-LSA (°)		61.4 ± 16.0	109.4 ± 9.9	< 0.001*
PI (°)		72.0 ± 12.1	57.3 ± 12.2	0.004*
PT (°)		39.8 ± 9.9	14.8 ± 5.7	< 0.001*
SS (°)		32.2 ± 14.6	43.1 ± 8.5	0.044
Pelvic orientation	Unbalanced pelvis	22(92%)	1(11%)	< 0.001* (Fisher’s exact test)
	Balanced pelvis	2(8%)	8(89%)	
LL (°)		57.7 ± 24.1	56.0 ± 11.5	0.787
TK (°)		5.4 ± 21.3	32.1 ± 9.6	< 0.001*
CL (°)		− 8.5 ± 16.1	10.5 ± 11.8	0.003*
Patients with cervical kyphosis		17(70.8%)	2(22.2%)	0.019* (Fisher’s exact test)
SVA (mm)		56.5 ± 35.1	36.2 ± 38.9	0.161
Patients with sagittal imbalance (SVA> 5 cm)		10(41.6%)	1(11.1%)	0.205

*Statistically significant *P* < 0.05

**Table 2:** Correlations between the spinal and pelvic sagittal parameters in patients with developmental spondylolisthesis (*n* = 33)

		Slip percentage	Dub-LSA	PI	PT	SS	LL	TK	SVA
Dub-LSA	Coefficient	− 0.780							
	*P* value	0.000							
PI	Coefficient	0.210	− 0.307						
	*P* value	0.241	0.082						
PT	Coefficient	0.498	− 0.789	0.523					
	*P* value	0.003	0.000	0.002					
SS	Coefficient	− 0.313	0.524	0.446	− 0.527				
	*P* value	0.076	0.002	0.009	0.002				
LL	Coefficient	0.387	− 0.078	0.260	− 0.248	0.524			
	*P* value	0.026	0.668	0.143	0.163	0.002			
TK	Coefficient	− 0.264	− 0.579	− 0.253	− 0.691	0.466	0.515		
	*P* value	0.137	0.000	0.156	0.000	0.006	0.002		
SVA	Coefficient	0.263	− 0.280	0.119	0.402	− 0.271	− 0.300	− 0.403	
	*P* value	0.138	0.115	0.509	0.020	0.127	0.090	0.020	
CL	Coefficient	− 0.200	− 0.446	− 0.346	− 0.592	0.272	0.162	0.683	− 0.115
	*P* value	0.263	0.009	0.048	0.000	0.126	0.367	0.000	0.523

Table 3 shows the comparative results between the preoperative and postoperative parameters in the HDDS group. The slip percentage was significantly decreased ([63.7 ± 25.5]% vs*.* [14.0 ± 17.0]%, *P* < 0.001). The postoperative Dub-LSA, PT, SS, and TK significantly differ from the corresponding preoperative parameters. There was no significant difference in CL at follow-up compared with that before surgery (− 3.4° ± 9.0° vs*.* − 8.5° ± 16.1°, *P* = 0.145). In total, 17 of the 24 HDDS patients had kyphotic cervical alignment (CL < 0°) before surgery. Interestingly, when we analyzed these 17 patients separately, postoperative CL was significantly increased compared with preoperative CL (− 16.4 ± 5.9° vs*.* − 3.6 ± 9.9°, *P* < 0.001). As mentioned above, a Dub-LSA less than 90° is considered significant lumbosacral kyphosis. Then, we divided the HDDS patients into two groups according to the postoperative Dub-LSA (< 90°, *n* = 13 vs. ≥ 90°, *n* = 11). The results showed that there was no significant difference in postoperative CL between the two groups (− 3.8° ± 8.3° vs. − 3.0° ± 10.1°, *P* = 0.83). And the Dub-LSA < 90° group had a higher proportion of patients with sagittal imbalance (SVA > 5 cm) than the Dub-LSA > 90° group after surgery (46% [6/13] vs*.* 0% [0/11], *P* = 0.016).

**Table 3:** Comparison between preoperative parameters and postoperative parameters in the HDDS group

	Pre-op (*n* = 24)	Post-op (*n* = 24)	*P* value
Slip percentage (%)	63.7 ± 25.5	14.0 ± 17.0	< 0.001*
Dub-LSA (°)	61.5 ± 16.0	83.6 ± 17.5	< 0.001*
PI (°)	72.0 ± 12.1	74.6 ± 11.5	0.197
PT (°)	39.8 ± 9.9	33.0 ± 9.3	0.003*
SS (°)	32.2 ± 14.6	41.6 ± 10.4	0.003*
LL (°)	57.7 ± 24.1	58.6 ± 11.2	0.856
TK (°)	5.4 ± 21.3	18.3 ± 12.7	0.001*
CL (°)	− 8.5 ± 16.1	− 3.4 ± 9.0	0.145
SVA (mm)	56.5 ± 35.1	35.5 ± 32.3	0.040*

*Statistically significant *P* < 0.05

## Discussion

The subjects of this study were all children and adolescents. Many studies have shown that there is a significant difference between pelvic parameters in this period of development and those of adults, and PI increases with age and then remains unchanged in adulthood due to maturity of the bone [4, 9]. Therefore, it is necessary to study adolescents and children separately from adults.

Labelle et al. [4] studied the relationship between pelvic parameters and the degree of spondylolisthesis in 214 young patients with developmental spondylolisthesis and concluded that a higher degree of spondylolisthesis was related to higher PI and PT. However, the authors did not distinguish patients with HDDS from those with LDDS. Our results showed that the PI and PT of the HDDS group were significantly higher than those of the LDDS group, indicating that the pelvic morphology and pelvic orientation of HDDS were different from those of low dysplastic lumbar spondylolisthesis. In this study, HDDS patients had a higher proportion (92%) of unbalanced retroverted pelvis according to Hresko et al.’s criteria [8]. Since adolescents with high-grade lumbar spondylolisthesis are usually diagnosed with HDDS, our results were consistent with Hresko et al.’s [8] conclusion that most patients with high-grade spondylolisthesis exhibited an unbalanced and retroverted pelvis.

**Fig. 2:**
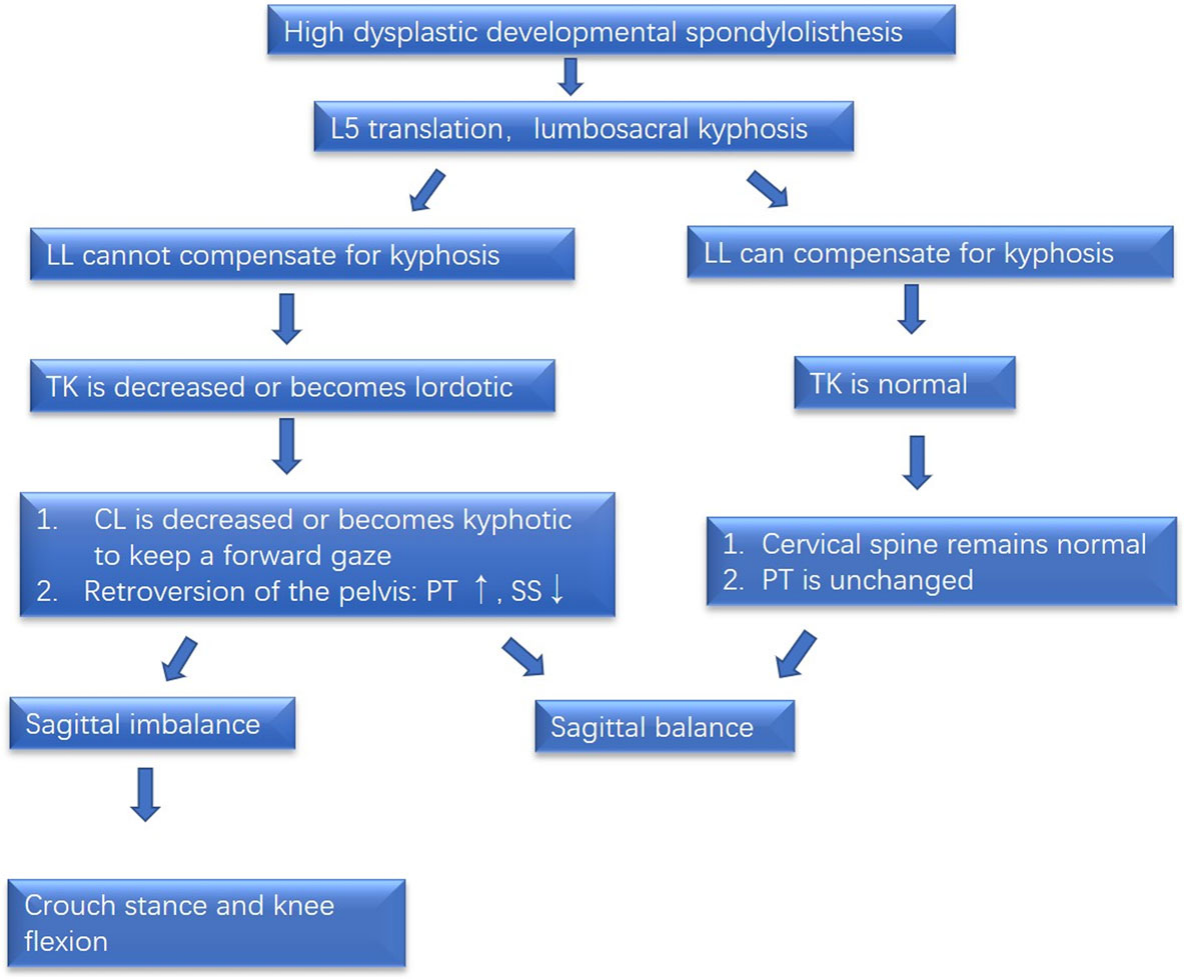
Spinal and pelvic compensatory mechanisms in HDDS patients

**Fig. 3:**
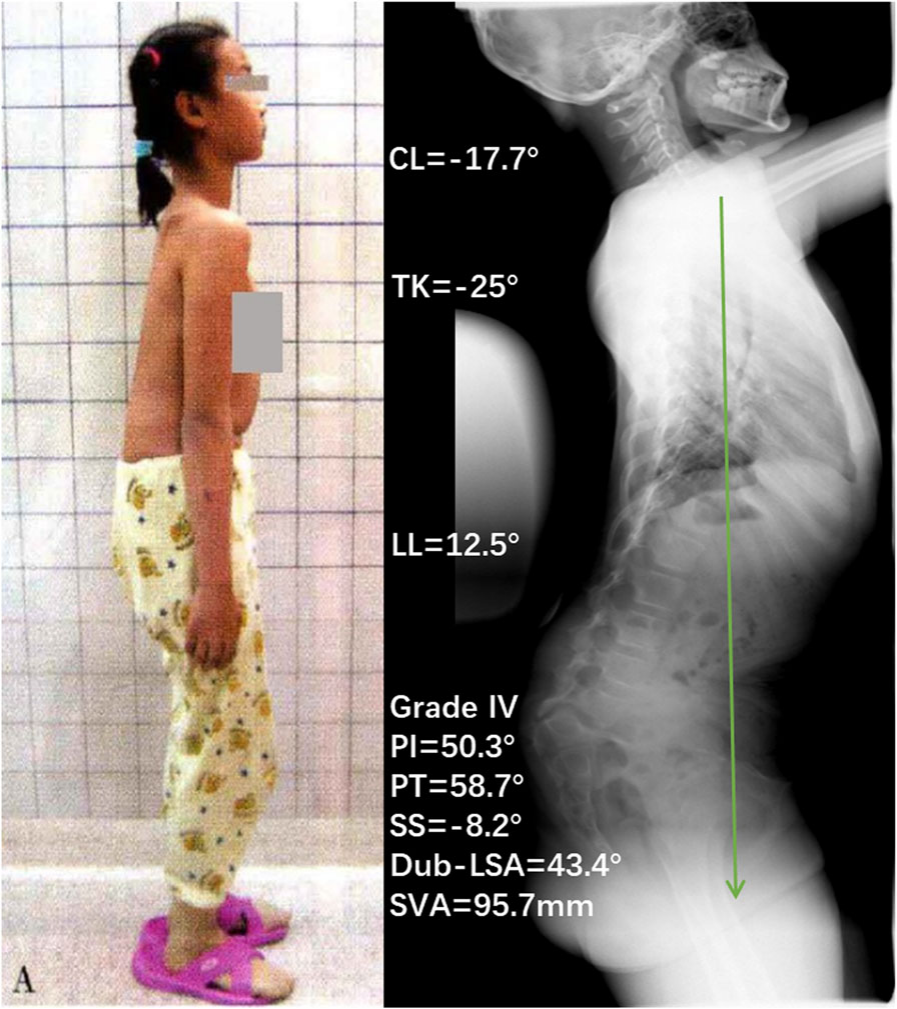
The possible mechanism of cervical kyphosis in patients with HDDS. A 10-year-old female with grade IV HDDS shows severe lumbosacral kyphosis (Dub-LSA = 43.4°); LL cannot compensate for kyphosis → TK becomes lordotic → CL becomes kyphotic to maintain a forward gaze; retroversion of the pelvis (PT ↑, SS↓) → if the spine is still unbalanced (the green arrow is C7PL), then knee flexion results in a crouched stance

**Fig. 4:**
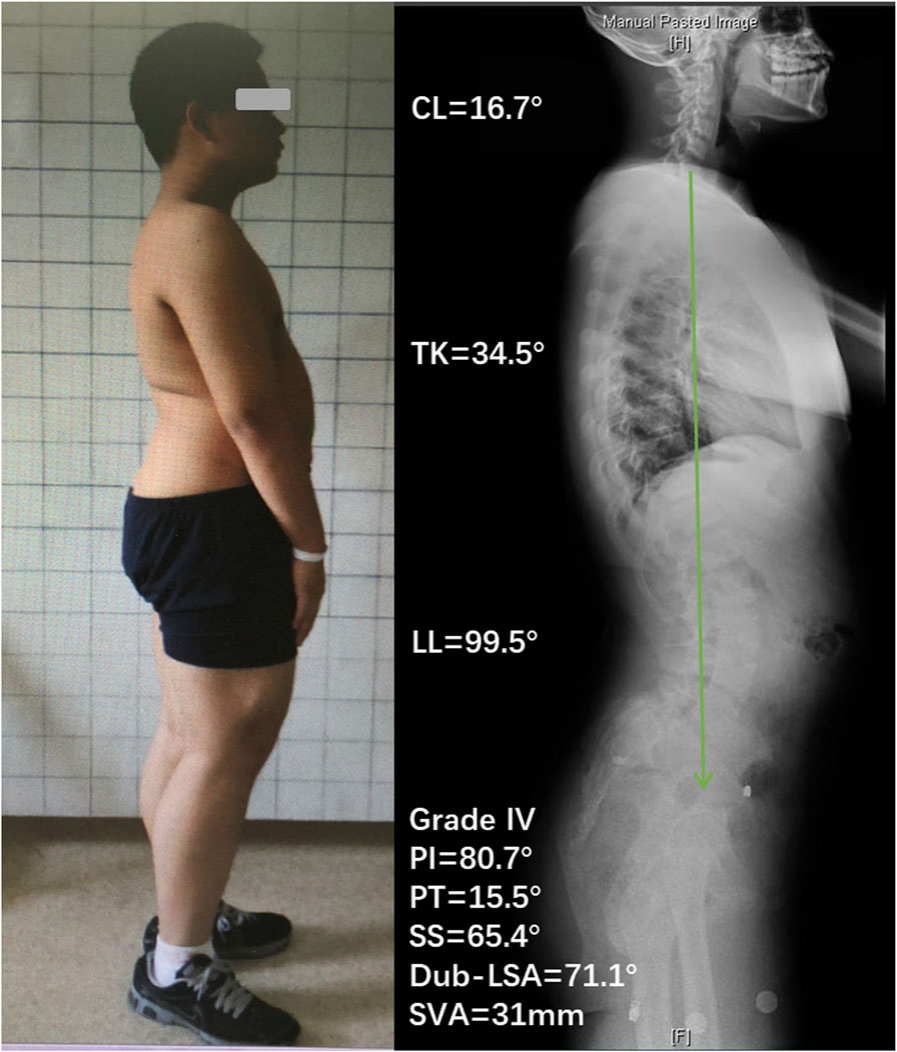
The possible mechanism of cervical lordosis in patients with HDDS. A 15-year-old male with grade IV HDDS shows significant lumbosacral kyphosis (Dub-LSA = 71.1°); LL can compensate for kyphosis → TK is normal → CL is lordotic, and the spine is balanced (the green arrow is C7PL)

Previous studies have shown that the cervical spine is mostly lordotic in the normal population, and CL in normal people is significantly related to TK but has no correlation with PI, LL, or SS [10, 11]. The results of this study showed that 70.8% of HDDS patients had kyphotic cervical alignment, a significantly higher proportion than that among LDDS patients (22.2%, *P* = 0.019), and TK in HDDS patients was significantly smaller than that of LDDS patients. Further, correlation analysis showed that CL in HDDS patients was correlated with lumbosacral kyphosis (Dub-LSA), PI, PT, and TK. Therefore, it can be inferred that the possible mechanism of cervical kyphosis in patients with HDDS might be as follows (Fig. 2): (1) HDDS leads to lumbosacral kyphosis. If LL fails to compensate for the lumbosacral kyphosis, TK will decrease or the thoracic spine may even become lordotic (exhibiting so-called “total spinal lordosis”) to balance the trunk. Then, CL will decrease or become kyphotic to maintain a forward gaze. In the most severe cases, if the spine is still unbalanced, the pelvis retroverts to its maximum degree, and the patient had to flex their knees to maintain an upright stance, resulting in a “crouched gait and stance” (Fig. 3). (2) If LL can compensate for lumbosacral kyphosis, then TK is still in the normal range, resulting in a lordotic or straight cervical spine and a relatively normal stance (Fig. 4). Gaines [12] described the mechanism of sagittal imbalance in patients with grade V L5 spondylolisthesis, which was similar to the first mechanism we mentioned above, but the author did not observe a relationship between cervical alignment and lumbosacral spondylolisthesis. Previous studies had shown that LL was increased in developmental spondylolisthesis patients [3, 4, 9]. However, our study showed that LL in patients with HDDS might increase or decrease. Whether LL increases or decreases depends on the patient’s own regulatory ability, which can also explain why our results show that LL has no correlation with the lumbosacral kyphosis (Table 2, *r* = − 0.078, *P* = 0.668).

**Fig. 5:**
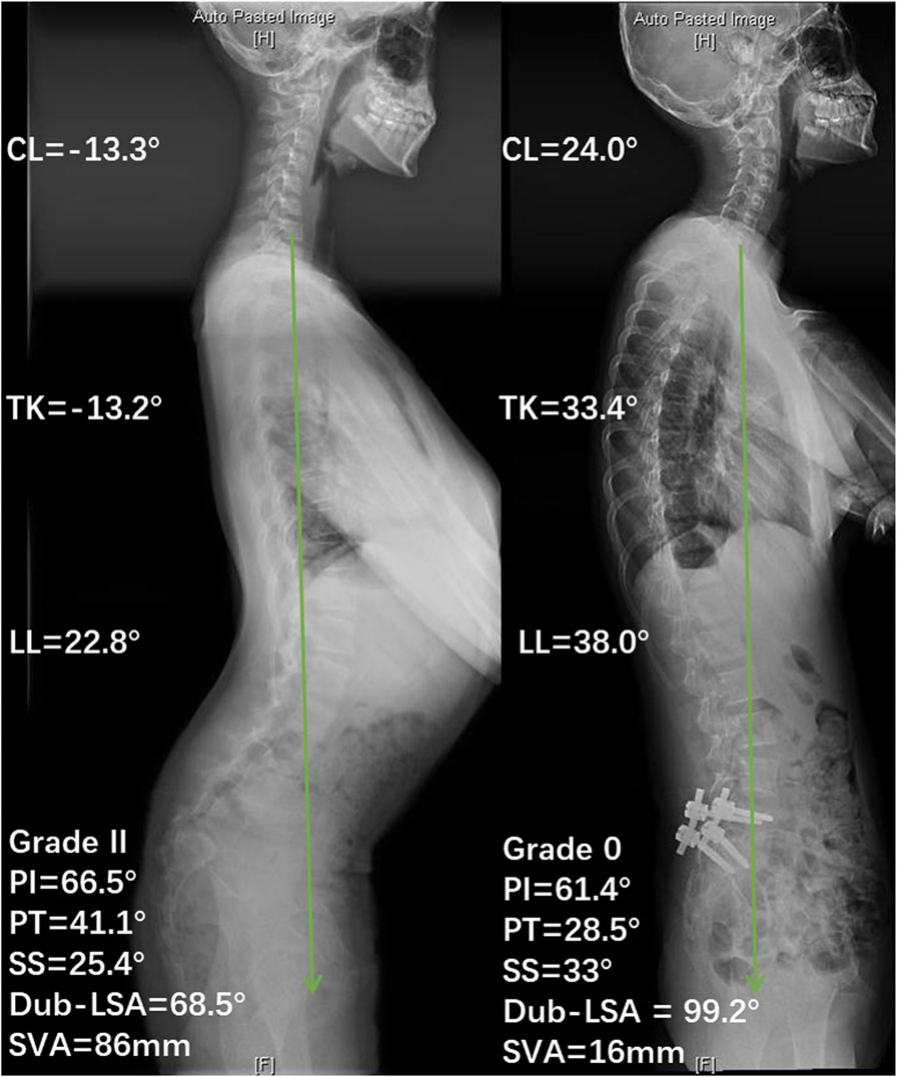
A typical case in which cervical kyphosis was corrected by reduction of spondylolisthesis. A 13-year-old female with grade II HDDS; the left is a preoperative image showing significant lumbosacral kyphosis (Dub-LSA = 68.5°), cervical kyphosis, and decreased TK, LL, and sagittal imbalance (the green arrow is C7PL). The right is a postoperative image with complete reduction, lumbosacral kyphosis corrected (Dub-LSA = 99.2°), CL becoming lordotic, and the whole spinal alignment has improved, as well as the sagittal balance (the green arrow is C7PL)

The unbalanced pelvis has a more severe lumbosacral kyphosis and a smaller TK, and Hresko et al. [8] suggest that reduction techniques might be considered in the unbalanced retroverted pelvic group, which supports performing reduction surgeries in patients with severe HDDS. Our results indicate that reduction of the slip and correction of the lumbosacral kyphosis for HDDS patients may normalize the overall spinal and pelvic sagittal profile (Fig. 5). In particular, CL in patients with kyphotic cervical spine was significantly improved after correction of the lumbosacral deformity (− 16.4° ± 5.9° vs*.* − 3.6° ± 9.9°, *P* < 0.001). Some studies showed that a kyphotic cervical curvature might be associated with neck pain [13, 14]. Although none of the HDDS patients complained of neck pain before or after surgery, however, long-term follow-up may be needed to observe these findings.

In the HDDS group, there were still 13 patients with Dub-LSA less than 90° after the operation, suggesting that the reduction of these patients was relatively poor. The results show there is no significant difference in postoperative CL between the poor and good reduction groups. The possible explanations may as follows: (1) The sample size is too small to produce a statistical difference. (2) The CL just has a moderate relationship with Dub-LSA (Table 2, *r* = − 0.446, *P* = 0.009), which the improvement of Dub-LSA may not correspond to the improvement of CL. (3) For the patients with poor reduction, their clinical effects are usually good if strong fusion and adequate decompression are achieved. These patients may adapt to the lumbosacral kyphosis through a series of compensatory mechanism, leading to an improvement of CL. At last, we cannot draw a conclusion that whether the extent of reduction corresponds to the extent of improvement in cervical curvature after surgery, which will be our next research direction.

Additionally, when a surgeon decides to reduce HDDS, what the objectives of the reduction procedure should be or how much reduction patients can tolerate remain unknown. Schwab et al. [15] suggest that the realignment objectives of adult spinal deformity should be patient-specific and involve attention to the following 3 parameters: SVA less than 5 cm, PT less than 25°, and LL proportional to the PI. However, the realignment objectives of ASD cannot apply in case of HDDS. For HDDS patients, many authors emphasized that the key point to improve the overall spinal alignment is to correct lumbosacral kyphosis rather than translation [16–18]. All the parameters of Dub-LSA, SDSG-dys-LSA, SDSG-LSA, etc. can well represent the degree of lumbosacral kyphosis [19, 20]. Among them, Dub-LSA is defined as the angle between the upper endplate of L5 and the posterior edge of the S1 vertebral body, and the two straight lines are relatively clear and not affected by the dome-like end plate of S1 or the trapezoidal shape of L5, making Dub-LSA easier to observe and measure than other parameters [6]. Glavas et al.’s [20] study showed that Dub-LSA has the best interobserver and intraobserver reliability and has the strongest correlation with the degree of slip percentage and slip grade. Dub-LSA is usually greater than 100° in normal people [19, 20]. If the Dub-LSA is less than 90°, lumbosacral kyphosis is considered significant [6]. We found that 46% of patients with postoperative Dub-LSA < 90° still had sagittal imbalance (SVA > 5 cm), while no sagittal imbalance was observed in patients with postoperative Dub-LSA > 90° (*P* = 0.016). Therefore, correction of Dub-LSA to above 90° might be acceptable and could be used as an objective to better improve the sagittal alignment of the spine in HDDS patients. However, the potential benefits of restoring sagittal spinal balance must be weighed against the risks of reduction, of which neurological deficits are the principal concerns.

This study has some limitations. First, it has the inherent limitations of a retrospective study, such as a relatively low level of evidence. Second, a small sample size and relatively short follow-up time are the main shortcomings, due to the rareness of the disease. Third, another limitation of this study is that standardized whole-spine x-rays are difficult to achieve. The technicians used different standards to make full-spine x-ray in different periods in our hospital. In some early cases, we used the method that Roussouly et al. [21] described in their 2005 article, which was the hands were placed on rests, and the patient was asked to stand in a comfortable but erect posture. In the other patients, we used the method described by Morvan et al. [22] in their 2011 article, which was standing both feet on the same alignment, 20–25 cm between the two feet, and upper arm fingers tip on the clavicle. Both methods were believed to have no impact on the spine. However, there is no study to compare these two methods. In recent years, the latter is becoming more widely used. Studies with larger sample sizes and more standardized whole-spine x-rays are still needed.

## Conclusions

To our knowledge, this is the first study to investigate the sagittal alignment of the cervical spine in patients with HDDS and to observe how the cervical spine responds to the reduction of spondylolisthesis. HDDS can lead to cervical kyphosis through a series of compensatory mechanisms. Reduction of spondylolisthesis and correction of lumbosacral kyphosis may correct cervical kyphosis and normalize the overall spinal sagittal profile. Correction of Dub-LSA to above 90° might be used as an objective to better improve the sagittal alignment of the spine.

## Data Availability

The datasets used and/or analyzed during the current study are available from the corresponding author on reasonable request.

## Abbreviations

HDDS: High dysplastic developmental spondylolisthesis
LDDS: Low dysplastic developmental spondylolisthesis
Dub-LSA: Dubousset lumbosacral angle
PI: Pelvic incidence
PT: Pelvic tilt
SS: Sacral slope
LL: Lumbar lordosis
TK: Thoracic kyphosis
CL: Cervical lordosis
SVA: Sagittal vertical axis
